# You Should Be the Specialist! Weak Mental Rotation Performance in Aviation Security Screeners – Reduced Performance Level in Aviation Security with No Gender Effect

**DOI:** 10.3389/fpsyg.2016.00333

**Published:** 2016-03-16

**Authors:** Jenny K. Krüger, Boris Suchan

**Affiliations:** Department of Neuropsychology, Institute of Cognitive Neuroscience, Ruhr-University BochumBochum, Germany

**Keywords:** computerized mental rotation test, aviation security screening, gender, training, shift work

## Abstract

Aviation security screeners analyze a large number of X-ray images per day and seem to be experts in mentally rotating diverse kinds of visual objects. A robust gender-effect that men outperform women in the Vandenberg & Kuse mental rotation task has been well documented over the last years. In addition it has been shown that training can positively influence the overall task-performance. Considering this, the aim of the present study was to investigate whether security screeners show better performance in the Mental Rotation Test (MRT) independently of gender. Forty-seven security screeners of both sexes from two German airports were examined with a computer based MRT. Their performance was compared to a large sample of control subjects. The well-known gender-effect favoring men on mental rotation was significant within the control group. However, the security screeners did not show any sex differences suggesting an effect of training and professional performance. Surprisingly this specialized group showed a lower level of overall MRT performance than the control participants. Possible aviation related influences such as secondary effects of work-shift or expertise which can cumulatively cause this result are discussed.

## Introduction

Mental rotation of objects is a fundamental spatial ability that affects several aspects of life as a basic cognitive function, for example parking a car in a parking spot accurately. According to Linn and Petersen’s (1985) meta-analysis, mental rotation has to be distinguished from spatial perception and spatial visualization (Voyer et al., 1995). Various influences on this ability, such as hormones (Hausmann et al., 2000; Piccardi et al., 2013) or sexual orientation (Peters et al., 2007) have been shown, as well as a robust gender-advantage where men tend to perform better than women (Linn and Petersen, 1985; Jordan et al., 2002; Cooke-Simpson and Voyer, 2007; Peters et al., 2007). Significant sex differences of mental rotation performance, especially characterized by the Vandenberg and Kuse (1978) Mental Rotation Test (MRT) has been shown (Linn and Petersen, 1985; Voyer et al., 1995). A vast majority of studies (e.g., see Suchan et al., 2006; Lippa et al., 2010; Foroughi et al., 2015; Zapf et al., 2015) on mental rotation abilities have been designed by using figural configurations such as the 3 dimensional (3-D) cubes conceptualized by Shepard and Metzler (Peters and Battista, 2008). Given that mental rotation provides the basis for recognition and categorization of visually perceived items, it seems reasonable that this competency should be very pronounced and well trained in aviation security assistants.

Aviation security is defined as the prevention of external risks (Yildiz et al., 2008), and is characterized by the critical interaction of humans and technology. Security assistants are responsible for controlling carry-on luggage and customers by performing analyses of X-ray images to detect prohibited objects, amongst other tasks (Singh and Singh, 2003). The increased importance preventing air traffic from attacks by terrorists involves new challenging duties for aviation security screening. Due to that change in endangerment the interpretation of X-ray images has been modified from mainly searching for typical weapons like knives or guns toward detecting of unconventional weapons like improvised bombs. Hence, screeners need a thorough knowledge of different types of threat categories (e.g., knives, bombs) and the components that are needed to compose highly threatening objects, e.g., Improvised Explosive Devices (IEDs). In order to detect and identify a prohibited object hidden in a complex carry-on luggage, aviation screeners must be capable of recognizing objects from all points of view, even if parts are overlapped by other objects, and the object is rotated over all possible angles. They have to be able to manipulate all of the perceived items mentally to make the correct decision (in this context: the luggage is safe or unsafe). Visual long-term memory and visual working memory, focused and divided attention, and especially mental rotation are all cognitive functions necessary to successfully perform valid X-ray interpretation (Krüger and Suchan, 2015). Screeners have to be able to compose a mental image of these 2 dimensional (2-D) X-ray images which can be compared with stored mental images of known objects. It has been shown, that visuospatial processing of 2-D matrices is associated with working memory related activity (Suchan et al., 2002). However, human memory is not typically capable of storing and retrieving each and every entity under all possible spatial circumstances (Shepard, 1967). As such, mental rotation and in this context the ability to recognize familiar objects even in unfamiliar settings and points of view is crucial for a high performance level in conducting security. Concerning the assumption that differences in spatial abilities are discussed as results of culturally based socialization (Goldstein et al., 1990) and gender-specific activities early on in life (Lauer et al., 2015), one can presume that working as an aviation security screener would eliminate these influences. In addition, it has been shown that specific training or daily spatial activities can enhance spatial abilities (Baenninger and Newcombe, 1995) and reduces gender-specific differences in mental rotation performance (Quaiser-Pohl et al., 2006). Taken together, aviation security screeners must be able to be highly focused while visually scanning the 2-D X-ray images, recognize, and categorize the shown items, and decide quickly whether there are any suspicious objects.

The current study focusses on the question whether security assistants can perform the mental rotation task at a high level without showing the well-known advantage of men. The idea behind this approach is that forming mental images of objects on a daily basis at work might result in an acquired expertise which eliminates the well-known gender differences. Given that screening X-ray images of carry-on luggage takes place normally on a computer screen, it is appropriate to conduct a computerized version of a complete mental rotation task. At present, there are only few studies that have used a fully computer-based version of Peters’ MRT, so there are currently no normative data available. To solve this issue, an online version of the computerized test was designed in order to collect data from control subjects. This webpage-based MRT was established on the same compilation of identical randomized items presented by Peters et al. (2007). The concern that online conducted questionnaires cannot afford the same data quality as lab-based cognitive assessments (McDonald and Stewart, 2003; Evans and Mathur, 2005; Noyes and Garland, 2008) is discussed controversially. Germine et al. (2012) demonstrated comparability between these two methods of investigation regarding diverse aspects, for example systematic patterns of anomalous data in performance, variance, and reliability, as well as effects of sample composition. Due to this, only technical aspects such as computer and internet expertise level or technical limitations (Reips, 2002) could have a realistic, critical impact on results in this type of research. Therefore, in addition to the examination of aviation security screeners’ ability to mentally rotate objects, data of a large online control sample was collected using the same task to establish a normative data-set for this new computerized version of the MRT.

## Materials and Methods

### Subjects

#### Aviation Security Screener

Fifty security screeners from two German airports performed the MRT. Participants were recruited from private security companies. Data were acquired in quiet rooms provided by the security company at the airports. Screeners participated either before or after their work shift and were reimbursed for participation (11 € per hour). The MRT was one of several tests of cognitive assessment which had a total duration of approximately 2 h depending on the participants’ working speed. The study was approved by the local Ethics Committee of the Psychological Faculty of the Ruhr – University Bochum. Participants provided their written informed consent to participate in this study. This consent procedure was approved by ethics committee. All participants had normal or corrected-to-normal vision. The age range was 20–60 years (*M* = 38.43 years, *SD* = 9.44), gender within the group was unequally distributed (women: 18, men: 29).

#### Control Groups

For reasons of comparison data of around 2,000 participants who were not professionally associated with aviation security screening were collected via the panel SoSci Survey (https://www.soscisurvey.de/mental_rotation/). These online participants were recruited via a variety of social networking sites, such as Facebook etc. The group of control participants performed the MRT online without restrictions of daytime. Comparable to other online questionnaires (Germine et al., 2012) distribution of participation with respect to gender was unequal (total number of female participants: 1,101, total number of male participants: 902) and in both groups participation was clearly pronounced in the age group 21–30 years and under 20 years of age.

##### Controls

In order to establish a comparable control group data set, participants were matched with respect to age and education. Due to the fact that the group of security screeners did not involve women with a university degree as highest level of education, all records of female control participants with a university degree were excluded prior to the main data analyses. In addition, the Anomaly Index was calculated separately for group and sex to indicate outliers or unusual cases (Schendera, 2010; for further information see Anomaly Index).

According to the security screeners, age range of the controls was 20–60 years (*M* = 30.95 years, *SD* = 10.32), gender within the group was unequally distributed (women: 557, men: 661).

##### Controls for Standardization

Participants of all classes of educational level, also woman with university degree, were included. Participants age ranged from 16 years to over 80 years (*M* = 28.90, *SD* = 10.96). Gender distribution of was unequal (total number of female participants: 1,054, total number of male participants: 828) and in both groups participation was clearly pronounced in the age group 21 to 30 years (women: 508, men: 388), followed by the number of participants under 20 years of age (women: 237, men: 153). After dividing the data in eight segments of 10 years (e.g., 21–30 years, 31–40 years) for the two gender groups separately, unusual cases were excluded by calculating an Anomaly Index. Outliers were defined as data which yielded an Anomaly Index > 2. At this one cluster was defined by gender and age-group (e.g., woman under 20 years). Partitioning participants’ data into the age groups separate for gender it became obvious that there were not enough completed MRT questionnaires for the age groups over 60 years. Due to this, results for these groups were not considered in the standard sample.

### Measures

#### Mental Rotation Task (Version A)

**FIGURE 1:**
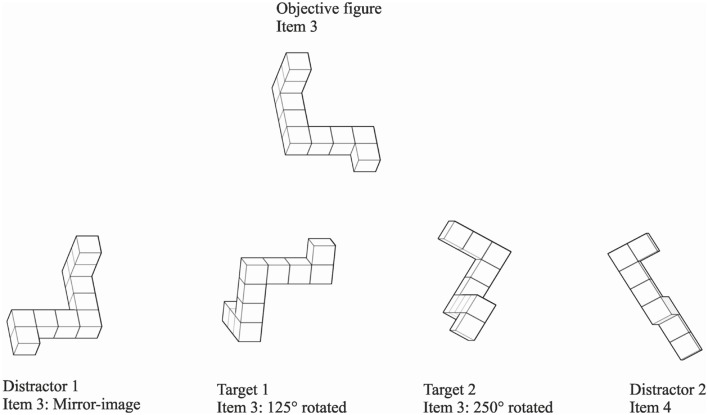
**Example for item selection based on invented rule number 1.** Items are arranged as used in the study. In addition to descriptive names as objective figure or target 1, item number and amount of rotation are given.

Using the MRT Library 2008 by Peters (Peters and Battista, 2008), a computer-based version of the MRT version A has been implemented. Peters’ stimulus library contains 4,627 images of 16 different figures and their mirror-images composed of cubes in line with the Shepard and Metzler’s approach. We choose only white cubes with black lines (**Figure 1**) in order to construct 24 trials, each containing five images for comparison. Comparable to the original paper–pencil version of the test (Vandenberg and Kuse, 1978), each trial included one objective figure and four figures as potential responses. Deviating from the original test, the objective figure was shown above the four response figures for reasons of limitations by the page-size. The response figures were presented side by side. All presented figures measured 140 × 140 pixels on a computer screen. Two of the four alternatives were rotated images of the original figure, and were identical in structure to the criterion figure (targets). The other two figures were incorrect alternatives, either an entirely different figure, or a mirror-image of the criterion figure (distractors). The MRT contained four blocks each of six trials and one practice block of four trials, comprising a total of 28 trials. Whereas test blocks did not involve feedback of performance, the previous practice block of four trials offered feedback in written and depicted form of the correct answers. Item selection was made on the basis of one of two experimenter-determined rules as described below (See Rules for Item Selection). These rules were constructed in order to ensure that the criterion figure and the four different selection figures were sufficiently distinct, and that none of the images had been picked out of the library twice by chance. Pre-testing brought up that three figures were too hard to be identified correctly due to their angle of presentation. These figures were replaced by another image of the same item that with a 5° difference in rotation. Beyond that, items number 1 and 2, as well the items 15 and 16 appeared to be almost structurally identical, and as a result they were utilized only for practice trials and as distractor figures. Further pre-testing showed that all remaining items could be perceived as distinct. Using this method, 140 images of the mental rotation stimulus library were selected and no image was used twice. Because of the required number of figures, items 3 through 14 were presented in two trials, but with different images.

Referring to Peters et al. (2007) testing time was limited to 10 min for all randomized 24 trials (25 s per trial), whereas the four practice trials were without any time-limit. A countdown timer in the top-right corner of the screen showed the remaining amount of time after the participants have started the test. In the case that the participants solved all trials under the given time, they had the opportunity to return and pass through all test trials to control and/or correct their selections. Otherwise, the task finished within the present trial when time-limit had been reached. Each trial was forced-choice, so the participants had to select two out of four response possibilities to proceed to the next trial. Participants were instructed in written form, that they have to click on two of the four alternatives, which they recognize as rotated versions of the objective figure. Within task instructions participants were asked to rotate shown images mentally and not by using head movements. To reduce guessing probability which could be increased by the forced-choice form, only two correct answers were scored with one point (Cooke-Simpson and Voyer, 2007). Correction for guessing seems to be very important given that woman might be more reluctant to guess in contrast to men (Goldstein et al., 1990; Voyer and Saunders, 2004).

#### Rules for Item Selection

The mental rotation stimulus library by Peters comprises 16 different items and their 16 mirror-images (version B of each item) which are stepwise rotated in stages of 5° (0° to 360°) around the x-axis, as well around the z-axis. The small-scale rotation around the two axis leads to 73 rotated figures per item for the x-axis and the same amount of figures for this item for the z-axis (in total 146 rotated figures per item). Bringing this together with the same amount of rotated figures for the mirror-image of each item (version B of the item) it comes in total to 292 different figures per item. Taken into account that the offered stimulus library contains this elaborate number of rotated figures of each item, item selection was conducted on the basis of one of two predefined rules. These rules were invented to ensure that not only very difficult or somewhat easy figures would be chosen out of the library by chance. Consequently, the objective figure and the four figures for selection were sufficient discriminative that the participants had to mentally rotate the shown objects to reach decision. Further, item selection by these rules ensured that none of the shown figures was used twice incidentally.

Partitioning the 24 test trials into 4 blocks (each block containing six trials) each rule was applied on two out of the four blocks. The first rule was used to provide item selection for the first and fourth block, whereas the second rule was disposed to the second and third block. The invented rules enumerate the angle differences of the four items for selection to the objective figure of each trial. For example (**Figure 1**) trial 1 consisted item 3 of the stimulus library in the original version rotated about 35° around the x-axis as the figure to compare with. Referring to the rule # 1 the two correct response figures (targets) belong as well to item 3, and one is 160° around the x-axis rotated (target 1), whereas the second correct choice is 285° around the z-axis revolved (target 2). Further, one of the two shown incorrect choices (distractors) is a mirror-image of item 3 in the same angle of presentation (here 35° of rotation around the x-axis; distractor 1), whereas the second one does belong to item 4 and is in addition in 85° revolved around the z-axis (distractor 2). For exact descriptions of the two rules see S1 Appendix A.

Furthermore, the selection of the items for the objective figure and their related selection figures was controlled as well. The selection of the objective figure was realized in steps of 15° rotation around x or z-axis (beginning with 5° of rotation). This means for the first block that the first trial involved item number 3 turned in 35° around the x-axis and following the second trial was realized by item 4 rotated about 50° over the x-axis. All following trials were created like this, whereas depending on the selected rule for the corresponding block the rotation of the target item was realized over the z-axis, as, e.g., for trial 7 item number 9 was chosen rotated in 125° over the z-axis.

### Data Analysis

#### Anomaly Index

Given that the distribution of the factors age, gender, and education between the two groups was heterogeneous, the Anomaly Index has been calculated to indicate unusual cases. This analysis was performed separately for each group (security screener and online control participants), which were further divided with respect to gender (e.g., female security screener) or for the standardization with respect to age (e.g., females 21–30 years of age). Anomaly Index offers information about the distance of every case (here overall performance of MRT) to the normal center of the cluster. The further away a case is from the group center, the more likely this case is unusual and has to be considered as an outlier. Each group (e.g., female security screeners) was treated as one cluster. Data of participants, which ranged above 2 SD (Anomaly Index value greater than 2) from the group mean performance, were excluded from further analyses. The calculation of the Anomaly Index segregated for each group was completed prior statistical evaluation of covariance for group difference to ensure that further analyses were contaminated by artifacts.

#### Mean MRT Performance

For the final analysis of the performance in the mental rotation task, data of 47 security screeners and 1,218 control participants were included and analyzed using IBM SPSS Statistics 20. A two-way analysis of covariance (ANCOVA) was conducted for the mean MRT performance including the factors group and gender with age in years and education in years as covariates. To control for influence of different sample sizes an additional one-way analysis of variance (ANOVA) including the factor gender was conducted for each group separately.

Special factors like duration of shift-work in hours, time of day (morning vs. evening) of the security screener group were not taken into account. Mental rotation performance was calculated as sum of all solved trials with one point for two right selections per item. This scoring procedure was chosen to further control for influence of guessing (Vandenberg and Kuse, 1978; Voyer et al., 1995; Peters et al., 2006). According to the number of trials 24 points could be reached. Likewise, reaction times were recorded for both groups.

## Results

### Mean MRT Performance

One-way analysis of variance (ANOVA) including the factor gender indicated a significant effect of this factor only within the control group *F*(1,1216) = 185.67, *p* < 0.001, η^2^ = 13.2%, power = 1.000 (α = 0.05) but not for the security screeners *F*(1,45) = 0.461, *p* = 0.501, η^2^ = 1.0%, power = 0.102 (α = 0.05).

**FIGURE 2:**
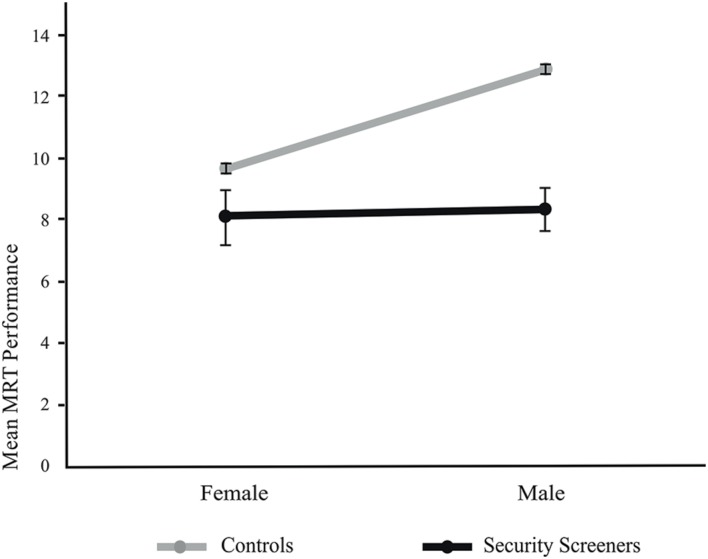
**Mental Rotation Test (MRT) performance (estimated mean and standard error; scoring out of 24) for controls and security assistants separated by gender.** Diagram is based on the a two-way analysis of covariance (ANCOVA) and to this controlled for the influence of the two covariates.

**FIGURE 3:**
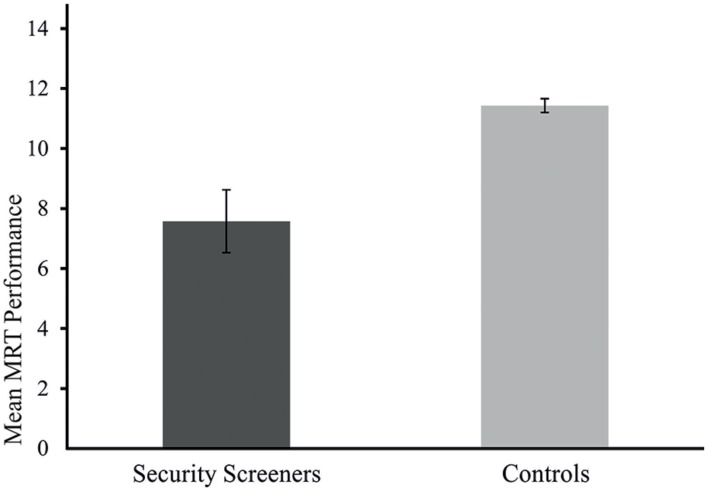
**Mean MRT performance (scoring out of 24) for controls and security assistants separately.** Errors bars depict 95% between-subjects confidence intervals of the mean performance for different sample sizes.

A two-way analysis of covariance including the factors group and gender and the covariate age and education was calculated. The statistical comparison yield evidence that the control group performed significantly better than the group of security screeners, *F*(1,1259) = 24.08, *p* < 0.001, η^2^ = 1.9%, power = 0.998 (α = 0.05). Levene’s test indicated equal error variances for both groups [*F*(3,1261) = 0.89, *p* < 0.45]. The average score of performance for control participants was 11.26 (*SE* = 0.11) and 8.42 (*SE* = 0.57) for security screeners. As can be seen in **Table 1**, a significant main effect for gender [*F*(1,1259) = 8.04, *p* < 0.005, η^2^ = 0.6%, power = 0.809 (α = 0.05)] could be shown and also the group gender interaction [*F*(1,1259) = 7.250, *p* < 0.007, η^2^ = 0.6%, power = 0.767 (*α* = 0.05)] reached significance (**Figure 2**). Group differences are depicted in **Figure 3.**

**Table 1:** Mental Rotation Test (MRT) performance (mean value and standard deviation) for the subgroups security assistants and normal controls divided by gender and in total by group.

	Subgroups					
	
	Security screeners		Controls			
		
	*N*	Mean	*SD*	*N*	Mean	*SD*
Females	18	7.11	3.51	557	9.68^∗^	3.83
Males	29	7.86	3.79	661	12.82^∗^	3.92
All participants	47	7.57^∗∗^	3.66	1218	11.43^∗∗^	4.16

Overall effect for Group *F*(1,1259) = 24.08, *p* < 0.001						
Overall effect for Gender *F*(1,1259) = 8.04, *p* < 0.005						
Overall interaction Group × Gender *F*(1,1259) = 7.25, *p* < 0.007						

Effect sizes for group, gender, and their interaction.						
^∗^*p* < 0.005; ^∗∗^*p* < 0.001.						


Within the covariance analysis all reported results were controlled for influences of the two covariates age and education. The covariate age in years was significantly related to mean mental rotation performance, *F*(1,1259) = 52.48, *p* < 0.001. Likewise, the covariate education in years showed a significant effect [*F*(1,1259) = 13.71, *p* < 0.001] on the overall performance in mental rotation.

The group by gender interaction was resolved using *post hoc* paired *t*-tests. Within the control group results suggest that men (*M* = 12.82, *SD* = 3.92) were performing better than women (*M* = 9.78, *SD* = 3.83; *t* = 13.63; *p* < 0.001). Contrary, there was no significant difference (*t* = –0.68; *p* < 0.501) for the factor gender within the security assistants (men: *M* = 7.11, *SD* = 3.51; women; *M* = 7.86, *SD* = 3.79). Comparisons between the groups regarding gender revealed significant difference (*t* = 2.92; *MD* = 2.67, *SE* = 0.92; *p* < 0.005) with female controls were performing better than female security screeners. This effect was even more pronounced for male control participants performing significantly better (*t* = 6.68; *MD* = 4.96, *SE* = 0.74; *p* < 0.001) than male security screeners. Differences between groups and gender are illustrated in **Figure 2.**

Between group comparisons of overall processed mental rotation tasks (*t* = –0.55, *p* < 0.588) and error rate in percent did not show significant differences (*t* = 0.48, *p* < 0.63).

Non-parametric correlation (Spearman’s Rho) between overall MRT solution rate and work experience in aviation security screening (in years) did not reach significance (*r*s = 0.045, *p* < 0.794, *n* = 36). Unfortunately, information regarding overall work experience in years independent of security company was not accessibly from the complete sample of aviation security screeners.

### Reaction Times

Two-way ANOVA including the mean of median reaction times did not show a significant difference between the two groups (*F* = 2.01, *p* = 0.156). Likewise, there was neither a significant interaction of sex within the groups (*F* = 1.45, *p* = 0.228) nor any interaction of sex and group (*F* = 1.35, *p* = 0.244).

### Standardization

After the removal of unusual cases using the Anomaly Index as described above data of 1,894 participants (age range < 20–60 years) were taken into account for standardization. The same segments of age range were used in order to define the distribution of MRT performance on a percentage basis as norm. To archive this, the percentile ranks of all obtained MRT scores were calculated for each age group separate for gender (e.g., males 21–30 years 12 points). As stated by Lienert and Raatz (1998, p. 291) percentiles were performed by two different statistical approaches to control for normal distribution. For an ideal normal distribution the results of percentile rank and cumulative frequency has to end in the same value. Calculation of normal distribution by statistical tests for normality as the Kolmogorov–Smirnoff are hardly reliable for large samples (*N* > 200), because they are too sensitive and would reach significance by even minute deviations. Comparisons of percentile rank and cumulative frequency as well as skewness led to rejection of normality for this huge sample of control participants. The transformation of percentiles in *z* or *t*-values of an abnormal dispersed sample would cause statistical distortions by smoothing the measured values and its proportions under the normal distribution. Percentile ranks are suitable as well for normal as also for anomalous distributed data. To avoid any distortions, the given results in S2 Appendix B (**Supplementary Tables S1** and **S2**) include solely scoring and corresponding percentiles.

## Discussion

The main aim of this study was to investigate mental rotation in specialists as defined by aviation security screeners. Mental rotation can be considered as being essential to perform work-related tasks as, e.g., interpretation of X-ray images. Valid X-ray interpretation for detection of unconventional threats and typical weapons is of major importance to assure high quality in security screening. IEDs can be composed by manipulation and combination of specific everyday objects. The targeted composition by certain objects can produce a highly threatening weapon like unconventional bombs. Consequently screeners have to recognize several parts of familiar objects and have to form connections between these unsuspicious objects. Contrary, typical weapons, e.g., fire-arms or knives can appear as black figures within X-ray image induced by their high density. Hence, it is reasonable to assume that standard weapons can be found more easily or produce a so-called ‘pop-out-effect’ and stop the visual search within an X-ray image. Correct interpretation of X-ray images is given by exact recognition of an object with its specific characteristics, its categorization followed by making the correct decision whether this object might be manipulated as a threat or not. Considering this, the basic cognitive ability to mentally rotate objects is fundamental for aviation security screening. For this purpose a complete randomized 24 trial computer based version of the MRT version A has been used in the present study. To our knowledge, this is the first computer based approach of a complete form of this MRT. Computerized mental rotation tasks included either just pairwise comparisons where participants had to decide whether two figures are identical (Voyer et al., 2006) or the tests did not involve the complete 24 test trials and the four practice trials (Reimers, 2007). Like in the paper–pencil version, participants had to manage the time they spent on each item with respect to its difficulty. Further, they had the opportunity to double-check their decisions when time permitted.

In addition to the investigation of acting security screeners, data of a large control group has been collected online. This data acquisition overcomes the shortcoming of standard sample for computerized MRTs and provides useful normative data for further investigations within diverse areas of psychological research.

Statistical analysis yielded two interesting and particularly surprising results. First, there was no gender difference within the group of security assistants with respect to their MRT performance. Secondly, contrary to our assumptions, security screeners performed less accurate than control participants. Interpretations of these results have to be limited due to the different sample sizes. However, analysis of variance can be accepted as a fairly robust method given homogeneity of variance even though the design is unbalanced. Combination of unequal sample sizes and violations of homogeneity of variance can lead to an underestimation of outcome differences which therefore results in non-significant effects (Field, 2009, p. 445). With respect to the present data this is not valid as homogeneity has been indicated by non-significant Levene-test. Furthermore, conducting ANOVA correction methods for imbalance (applying Sums of Squares Type III) can be seen as a rather conservative procedure in indicating significant interaction effects. Within this procedure adjustments are given for both variables and the interaction (Landsheer and van den Wittenboer, 2015; Shaw and Mitchell-olds, 2015). Even though, comparisons of unequal sample sizes lead to controversy discussion. Based on this, we verified the demonstrated effects in two ways by calculation separate univariate ANOVAs per group and an ANCOVA for both groups together.

Next, we like to discuss the surprising finding that security screeners did not perform better on mental rotation than controls. Results will be clarified with respect to possible explanation and influences on the low level of performance. Proposed interpretation of results from a correlative study cannot finally distinguish the impact of several factors or interactive influences.

As expected, security screeners did not show the well-established gender difference. A comparable finding has been shown by Verde et al. (2013) investigating pilots and non-pilots regarding different spatial abilities. For mental rotation performance they could show that the gender-effect on the speed of cognitive spatial processing was not given within the group of professional pilots but for the controls. In line with the reported results by Dror et al. (1993) pilots were significantly faster than non-pilots. However, overall group or gender differences with respect to accuracy could not be reported. This is particularly surprising because it can be assumed that pilots have enhanced spatial navigation skills (Sutton et al., 2014) which are related to mental rotation. Furthermore, gender differences for spatial navigation in terms that men tend to perform better has been shown as well (Palermo et al., 2008). Though, these results are in line with studies which have shown that spatial training has a positive influence on mental rotation performance and reduces gender-based differences (Quaiser-Pohl et al., 2006; Debarnot et al., 2013). Thus, comparable solution rates of male and female security screeners can be explained as a result of their everyday exposure to mental rotation of diverse visual figures during work. Barrientos (2000) could show that aviation security assistants need approximately about 15 s for analyzing an X-ray image (8.5 s for scanning and 6.5 s for the decision if a bag needs further inspection or not). Hence, security assistants analyze a very large number of X-ray images through 8–10 h work-shift. Conventional X-ray images are 2-D images of 3-D real-world objects (Yildiz et al., 2008). Due to this, security screeners should be very accustomed to 2-D pictures whereas 3-D pictures can be considered as rather novel to them. This is especially important as the cubes used in the MRT are defined as 3-D stimuli (Suchan et al., 2006; Neubauer et al., 2010). Furthermore, it has been shown that male perform better with 2-D presentations. Neubauer et al. (2010) indicated that the effect of training on mental rotation performance is differentiated by the use of 2-D versus 3-D objects. Regarding the absence of a gender-effect within the group of adolescent participants for the training with 3-D stimuli, they suggest that male participants had already prior training higher level of mental rotation abilities that is restricted to 2-D presentations. Therefore males performed better in 2-D rotations before and after training in comparison to the female participants. But this advantage was not the case for the 3-D rotations because prior experience in this task was comparable for males and females. Hence, female participants enhanced their rotation skills by training in an equal manner as the males. This result is supported by finding showing that the neuronal mechanisms of processing of 2-D and 3-D objects differ with respect to their spatial features (Suchan et al., 2006). Regarding the result that male and female security screeners perform on the same level in 3-D mental rotations it is possible that familiarity and training for certain spatial dimensions (2-D vs. 3-D) may generalize between spatial dimensions. This interpretation is in line with the clear gender-effect within the control sample that had no particular training, neither for 3-D nor for 2-D objects. Voyer et al. (1995) claim that decreased mental rotation performance by females is moderated by difficulties in combining different spatial dimensions (‘dimensionality crossing’). One can argue that the generalization of training between the dimensions is moderated by less difficulties due to ‘dimensionality crossing’ within the female security screeners. Given that security screeners have to manipulate 2-D images of 3-D real objects on a daily basis, it seems reasonable that this leads to enhanced abilities in ‘dimensionality crossing’. Security screeners have to analyze images of real luggage. In suspicious situations they have to conduct additional, manual inspection whether the carry-on bag contains a forbidden item. Within this scenario, screeners have to compare 2-D images with 3-D real objects.

This combination could cause the elimination of the gender-effect within the MRT. Considering this, it seems possible that comparable mental rotation performance of male and female security screeners is caused by the same level of experience due to practice during everyday work. Hence it can be stated that security screeners have developed a certain expertise within mental rotation of rather unknown objects. The extensive discussion of Bilalić et al. (2008) on expertise and related disadvantages is very promising for that idea. The authors reviewed and demonstrated within a series of experiments how experts of different level can become inflexible in finding new, creative solutions for a given problem. Expertise is stated as developed by thorough knowledge for certain aspects and consolidation of specific problem-solving strategies. Due to that, expertise is a result of automatization and can lead to inflexibility induced by prior knowledge that results in a blocking effect of the familiar solution. But this finding is only true for a certain level of expertise. With increasing expertise the blocking effect decreases and finding new, creative solutions for problems being considered as familiar become reachable again. Given that, security screeners should be experts on a medium level that can cause inflexibility in recognizing better solutions if the task is considered as known. Furthermore, the authors could support their idea of different stages in becoming an expert within a functional imaging study Bilalić et al. (2014). Within a 1-back-task they compared medical students with experienced radiologists on domain specific (here thorax X-ray images) and non-specific images. The authors could demonstrate differences in performance and specific neuronal activation patterns within the fusiform gyrus due to stimuli from domain specialization and outside this domain. Unfortunately, Bilalić et al. (2014) did not include a completely unexperienced control group to compare differences in task performance and to find out whether stepwise changes in fusiform gyrus activation is related to particular stages in the development of expertise.

Furthermore, several influences on the performance of the security screeners have to be considered. It is plausible that different factors together may cause the low overall solution rate. Primarily, the two groups differ with respect to the duration they had to solve the cognitive tests. Security screeners had to complete 2 h of cognitive assessments, whereas the controls spent only 20 min on solely the MRT. Both groups were self-selected by the meaning that the participation was voluntarily. One could argue that security screeners had a higher level of motivation caused by payment, whereas controls did not receive any money for their involvement. On the other hand, screeners had to make an appointment in advance, for which they had been encouraged to keep. Control participants were totally independent in their choice when and where they would like to realize the MRT. In addition to the fact that screeners did not perform the test to a self-selected moment in their leisure time, they conducted the cognitive assessment more or less directly before or after work shift. Linked to this, special aviation referring factors like duration of shift-work in hours, time of day (morning versus evening) can be considered to influence the level of performance. Information of the mentioned aspects were not taken into account as factors in the statistical analyses because these were not completely available for the control group that performed the task online on their own. However, screeners indicated via visual analogue scales their subjective state of concentration, motivation and attention, and also their sleepiness via the Stanford Sleepiness Scale (Hoddes et al., 1972). Results identified effects of time (morning versus evening) and time of testing (before versus after work shift). Thus, screeners declared an increase of sleepiness and a depression of all further mental factors with the passing of time. In this context, Basner et al. (2008) demonstrated in a simulated X-ray screening task the adverse influences from shift work on threat detection accuracy, compound by hit rate, false alarm rate, detection accuracy, and response bias. They yielded evidence that night work and related to that sleep loss disadvantageously affect the accuracy in detecting complex real world objects. Beyond that, the negative influence of shift work is well documented for the circadian rhythm and the resulted difficulties at maintaining and initiating sleep (Akerstedt, 1990, 2003). Disruptions in the sleeping cycle induce adverse effects on cognitive functions as working memory, attention, executive functions (Rouch et al., 2005; Marquié et al., 2014). Referring to the poor performance of the security screeners, it is possible that the performance level was also affected by the negative influences of sleep deprivation. This suggests that the overall solution rate is affected by many other factors, e.g., shift work and training which is true for both sexes. This interpretation is supported by the study of Debarnot et al. (2013) who have investigated the influence of sleep on mental rotation training. Here they demonstrated that women and men perform significantly better in the MRT after a night of sleep. While a similar period of resting without sleep during the day led to a decrease of mental rotation performance. Additionally they could show that women enhance their performance level by training as men do. Thus, the demonstrated beneficial effect of sleep on mental rotation training independently of gender can be considered as part of the explanation for the reported findings.

Further, potential bias in self-selection refers to the time the person spends with computer activity (Evans and Mathur, 2005). Practice and familiarity in contact and operating a computer as well as several software programs provide a confident handling of these (Reips, 2002). While assessing the cognitive abilities of the security screeners via different testing systems, half of the used tests were computerized, the impression consolidated that most of the participants of these group had little to none experience in working with a computer. They were well instructed in written form and had personal assistance in case of any issues with the tasks. Even though it can be assumed that the limited computer practice has an attenuating impact of the overall performance. On the other hand, it can be supposed that the self-selected normal control group recruited via different webpages is more likely to work with a computer. Further, Terlecki and Newcombe (2005) argue that especially differences in gender regarding experience with computer and video gaming may contribute to difference in spatial abilities as mental rotation. However, typical influence of computer-use like excessive computer gaming experience could not be considered because it was not assessed via the online survey. But for all that, Voyer et al. (2006) demonstrated in a validation study no disparity between paper-and-pencil and a computerized mental rotation tasks in measuring the underlying ability to rotate 3-D objects quickly and accurately in mind. Together, we cannot completely rule out that neither becoming an aviation security screener nor self-selection effects due to participating in this study within both groups, can lead to certain selection biases which possibly influence the described results. Furthermore online participants could not be controlled for deviating behavior for instance hand or head movements in order to facilitate spatial rotation of the shown items.

In this context Peters et al.’s (2006) discussion about possible influences on the overall solution rate by self-selection and possible changes in the magnitude of the gender-effect referring the level of performance is very encouraging. In their report, they compared student’s ability for mental rotation from different academic programs (science and engineering versus arts and social science). Against given expectations they could not indicate an interaction between sex and academic program whereby the correlation between spatial ability and scientific as well as mathematical reasoning is substantial for this assumption (Hegarty and Kozhevnikov, 1999). It is imaginable that only women who have strengthened mathematical or visual–spatial skills decide for a study in natural science, whereas if this is not the case another subject of study would be chosen. Due to this effect of self-selection, Peters et al. (2006) postulate an inverted U-function for the gender-effect where on both ends the difference diminishes in overall performance between male and female participants. Based on this assumption the failed gender-effect within the group of security screeners can be considered as caused by the baseline effect, where the scores are too close to minimum to differentiate between the sexes anymore. Anyway, one could argue that an equal level of performance for male and female aviation security screeners indicates an effect of training. Considering the results demonstrated on the advanced mental rotation abilities of pilots (Verde et al., 2013), one could assume that both groups represent the end of an U shape distribution where the gender effect diminishes. However, the advantage of pilots refers to the reaction time and not to the overall mental rotation performance. With respect to accuracy there was no overall significant difference between the groups and gender. Although the investigation by Verde et al. (2013) on the specialized group is of particular interest, this study cannot be compared to the current study directly. In contrast to our study, mental rotation performance was not examined with the classical/psychometric Vandenberg & Kuse task, which has been verified within meta-analysis (Voyer et al., 1995) and factorial analysis (Voyer and Saunders, 2004) to produce the largest and most reliable gender-effect. Further, the advanced mental rotation abilities in pilots were mainly demonstrated by decreases in reaction times. However, it has been proposed that for meaningful interpretations of differences in reaction times it seems reasonable to analyze the slope of reaction times in relation to the rotation angle or in relation to accuracy (Linn and Petersen, 1985; Debarnot et al., 2013; Zapf et al., 2015). Taken this into account, interpretations of the reported non-significant difference in reaction times between controls and security assistants might have some limitations. Nevertheless, there were neither significant differences between the groups nor any sex differences within each group. Though this finding is consistent with the results reported by Neubauer et al. (2010) and Debarnot et al. (2013) that sex differences occurred solely in mental rotation scores but did not reach significances for reaction times. Furthermore, the computerized test was not mainly conducted to measure pure reaction times of particular trials. The recorded time for each trial is the duration of dwell, which includes the time the participant needed to solve the task, to select the made choices by clicking on the button of each figure and to click on the “next” button to proceed to the following trial. Additionally for the control group processing time caused by capacity of the internet browser could have had a negative impact on the documented reaction time. In this context reaction time measurements cannot be considered as very meaningful referring the question of performance level. Due to the claim of completeness the calculated analysis of reaction time were reported and discussed here.

## Conclusion

The present study aimed to investigate the basic ability to mentally rotate figures within the very specific participant group of aviation security screeners in comparison to a normal control group. Statistical analysis yielded partially unexpected results that raise several questions regarding different external influences such as shift work on task performance, as well as the personal aspects as, e.g., familiarity with computer use, within both groups. As discussed above an effect of training cannot be assumed exclusively as several influences of selection cannot be completely excluded. As mentioned by Peters (Peters et al., 2006) it is imaginable that only women with enhanced spatial abilities decide to work in aviation security screening or otherwise that only those stay working within security screening over years who have effectively developed enhanced mental rotation abilities. It is worth to mention that these arguments could be true for several applied investigations, for example how training increases hippocampus volume by taxi drivers (Maguire et al., 2006).

Even regarding the described limitations, this investigation generated very promising results by data of a large control sample which can be consulted in sense of comparison as standard sample including details to age, education and gender for a computer based MRT (version A). Furthermore, the empirical analyses of actual working security screeners clarified the importance of cognitive assessments in staff selection as well as additional cognitive trainings and provoked questions in respect to cognitive impairments induced by general working framework and uneven work-life-balance.

## Author Contributions

All authors listed, have made substantial, direct, and intellectual contribution to the work, and approved it for publication.

## Conflict of Interest Statement

The authors declare that the research was conducted in the absence of any commercial or financial relationships that could be construed as a potential conflict of interest.
