# Supplementary material for: You Should Be the Specialist! Weak Mental Rotation Performance in Aviation Security Screeners – Reduced Performance Level in Aviation Security with No Gender Effect
Source: Front Psychol. 2016 Mar 16;7:333. doi: 10.3389/fpsyg.2016.00333 (PMC4792886; doi:10.3389/fpsyg.2016.00333)
Supplement: Supplementary file 1 [file Table_1.DOCX]

# S1 Appendix A

Rule 1 (Block 1, Trial 1 - 6 and Block 4, Trial 19 - 24):

Target 1 has been rotated about 125° in the same axis (as the objective figure), while target 2 is in 250° turned in the other axis.

Distractor 1 is the precise mirror-image, while distractor 2 belongs to the next item of the library and is additionally 50° rotated in the other axis.

Rule 2 (Block 2, Trial 7 – 12 and Block 3, Trial 13 - 18):

Target 1 is rotated about 125° in the other axis as the objective figure is. The target 2 is turned about 250° in the same axis.

Distractor 1 is a mirror-image, which is revolved in the other axis about 50°. Distractor 2 is part of the next item and is additionally 50° rotated.
