# Supplementary material for: You Should Be the Specialist! Weak Mental Rotation Performance in Aviation Security Screeners – Reduced Performance Level in Aviation Security with No Gender Effect
Source: Front Psychol. 2016 Mar 16;7:333. doi: 10.3389/fpsyg.2016.00333 (PMC4792886; doi:10.3389/fpsyg.2016.00333)
Supplement: Supplementary file 2 [file Table_2.DOCX]

# S2 Appendix B

# S2 Table 1 Scoring of mental rotation performance and corresponding percentiles for male participants of age range under 20 years until 60 years (in 10 years) as standard sample (n = 816).

|  | Corresponding percentiles of scoring for age range (in 10 years) | | | | |
| --- | --- | --- | --- | --- | --- |
| Scoring | **< 20**  **(n = 153)** | **21 – 30**  **(n = 388)** | **31 – 40**  **(n = 135)** | **41 – 50**  **(n = 90)** | **51 – 60**  **(n = 50)** |
| 1 | * | * | * | * | * |
| 2 | * | * | * | * | * |
| 3 | * | * | * | * | * |
| 4 | * | * | 5 | * | 4 |
| 5 | 1 | * | 10 | 6 | 8 |
| 6 | 3 | 3 | 12 | 12 | 12 |
| 7 | 5 | 5 | 18 | 21 | 28 |
| 8 | 6 | 10 | 31 | 36 | 36 |
| 9 | 11 | 15 | 27 | 48 | 50 |
| 10 | 16 | 19 | 35 | 63 | 70 |
| 11 | 25 | 25 | 48 | 71 | 80 |
| 12 | 31 | 32 | 54 | 80 | 84 |
| 13 | 40 | 44 | 60 | 90 | 86 |
| 14 | 47 | 53 | 67 | 93 | 92 |
| 15 | 60 | 63 | 76 | 99 | 94 |
| 16 | 63 | 71 | 93 | 100 | 100 |
| 17 | 73 | 81 | 93 | * | * |
| 18 | 85 | 86 | 97 | * | * |
| 19 | 89 | 93 | 99 | * | * |
| 20 | 96 | 97 | 100 | * | * |
| 21 | 100 | 100 | * | * | * |
| 22 | * | * | * | * | * |
| 23 | * | * | * | * | * |
| 24 | * | * | * | * | * |

Note 1 * indicates no available data for this particular age range and scoring.

# S2 Table 2 Scoring of mental rotation performance and corresponding percentiles for female participants of age range under 20 years until 60 years (in 10 years) as standard sample (n = 1043).

|  | Corresponding percentiles of scoring for age range (in 10 years) | | | | |
| --- | --- | --- | --- | --- | --- |
| Scoring | **< 20**  **(n = 237)** | **21 – 30**  **(n = 508)** | **31 – 40**  **(n = 154)** | **41 – 50**  **(n = 106)** | **51 – 60**  **(n = 38)** |
| 1 | 1 | * | * | 2 | * |
| 2 | 3 | * | * | 4 | * |
| 3 | 6 | 4 | 3 | 10 | 5 |
| 4 | 9 | 6 | 6 | 14 | 11 |
| 5 | 11 | 10 | 12 | 17 | 24 |
| 6 | 13 | 15 | 18 | 22 | 42 |
| 7 | 18 | 22 | 31 | 35 | 63 |
| 8 | 27 | 30 | 40 | 44 | 71 |
| 9 | 36 | 41 | 50 | 53 | 90 |
| 10 | 46 | 48 | 63 | 63 | 97 |
| 11 | 55 | 57 | 70 | 72 | 100 |
| 12 | 67 | 65 | 79 | 76 | * |
| 13 | 73 | 75 | 86 | 82 | * |
| 14 | 80 | 79 | 92 | 88 | * |
| 15 | 84 | 86 | 97 | 94 | * |
| 16 | 80 | 94 | 100 | 97 | * |
| 17 | 92 | 98 | * | 99 | * |
| 18 | 96 | 100 | * | 100 | * |
| 19 | 98 | * | * | * | * |
| 20 | 99 | * | * | * | * |
| 21 | 99 | * | * | * | * |
| 22 | 100 | * | * | * | * |
| 23 | * | * | * | * | * |
| 24 | * | * | * | * | * |

Note 2 * indicates no available data for this particular age range and scoring.
